# Nitrifying Communities in Biological Nitrogen Removal Processes at Tropical Municipal Wastewater Treatment Plants

**DOI:** 10.1264/jsme2.ME25036

**Published:** 2025-09-10

**Authors:** Liang Feng, Jia Xing Loi, Joana Séneca, Petra Pjevac, Faidzul Hakim Adnan, Gek Cheng Ngoh, Bee Chin Khor, Alijah Mohd Aris, Mamoru Oshiki, Holger Daims, Adeline Seak May Chua

**Affiliations:** 1 Sustainable Process Engineering Center, Department of Chemical Engineering, Faculty of Engineering, Universiti Malaya, 50603 Kuala Lumpur, Malaysia; 2 Division of Microbial Ecology, Centre for Microbiology and Environmental Systems Science, University of Vienna, 1030 Vienna, Austria; 3 Joint Microbiome Facility of the Medical University of Vienna and the University of Vienna, 1030 Vienna, Austria; 4 Indah Water Konsortium Sdn Bhd, No. 1, Jalan Damansara, 60000 Kuala Lumpur, Malaysia; 5 Division of Environmental Engineering, Faculty of Engineering, Hokkaido University, 060–8628 Hokkaido, Japan; 6 The Comammox Research Platform, University of Vienna, 1030 Vienna, Austria

**Keywords:** ammonia-oxidizing archaea, ammonia-oxidizing bacteria, comammox, nitrification, tropical wastewater treatment plants

## Abstract

Nitrifying communities in activated sludge play a crucial role in biological nitrogen removal processes in municipal wastewater treatment plants. While extensive research has been conducted in temperate regions, limited information is available on nitrifiers in tropical regions. The present study investigated all currently known nitrifying communities in two full-scale municipal wastewater treatment plants in Malaysia operated under low-dissolved oxygen (DO) (0.2–0.7‍ ‍mg‍ ‍DO‍ ‍L^–1^) or high-DO (2.0–5.5‍ ‍mg‍ ‍DO‍ ‍L^–1^) conditions at 30°C. The core nitrifiers in the municipal wastewater treatment plants were *Nitrosomonas* (ammonia-oxidizing bacteria, AOB), *Nitrospira* (nitrite-oxidizing or complete ammonia-oxidizing, comammox, bacteria), and ammonia-oxidizing archaea (AOA) as identified by a 16S rRNA gene amplicon sequencing ana­lysis and corroborated by 16S rRNA-targeted fluorescence *in situ* hybridization. A quantitative polymerase chain reaction (qPCR) targeting ammonia monooxygenase subunit A (*amoA*) genes revealed stable populations of comammox *Nitrospira* and AOB in both wastewater treatment plants. AOA were detected in only one of the plants and their population sizes fluctuated, with higher temporary abundance under high-DO conditions. These results provide important insights into the composition and dynamics of nitrifying communities in tropical municipal wastewater treatment plants.

Nitrogen pollution from municipal wastewater is one of the key factors contributing to water eutrophication (Chen *et al.*, 2020), and nitrogen removal at municipal wastewater treatment plants (WWTPs) is critical for reducing their impact on natural water ecosystems. The nitrification-denitrification process has been widely used for biological nitrogen removal from wastewater (Winkler and Straka, 2019), in which NH_3_ is aerobically oxidized to NO_3_^–^ by nitrifiers, and formed NO_3_^–^ is then reduced to N_2_ gas by denitrifiers (Tchobanoglus *et al.*, 2003). Nitrification is the rate-limiting step in the biological nitrogen removal process (Jaramillo *et al.*, 2018), and a diverse functional group of microorganisms, including aerobic ammonia-oxidizing archaea (AOA), ammonia-oxidizing bacteria (AOB), nitrite-oxidizing bacteria (NOB), and complete ammonia-oxidizing (comammox) bacteria are involved in nitrification (Daims *et al.*, 2015; Van Kessel *et al.*, 2015; Ren *et al.*, 2020).

The community composition of nitrifiers and their abundance in activated sludge have been investigated using culture-dependent and -independent methods (Daims *et al.*, 2001a; Cai *et al.*, 2018). The MiDAS project exami­ned microbial community structures in >740 WWTPs globally, and identified *Nitrosomonas* and *Nitrospira* as core nitrifying bacteria commonly present in wastewater treatment processes (Dueholm *et al.*, 2022). In a global study on activated sludge from 269 WWTPs across 23 countries on six continents, *Nitrospira* was identified as a core taxon, highlighting its critical role in nitrite oxidation or complete ammonia oxidation (Wu *et al.*, 2019). While global surveys provide valuable snapshots of microbial community structures in WWTPs, long-term monitoring surveys have clarified the dynamics of microbial community structures and the impact of reactor operational conditions (Muszyński *et al.*, 2015); *i.e.*, dissolved oxygen (DO) concentrations and temperature fluctuations had a strong impact on population dynamics (Johnston *et al.*, 2019). DO is a key environmental factor shaping the composition of nitrifying communities and the efficiency of nitrification because of differences in the oxygen affinities of nitrifiers. For example, *Nitrospira* species exhibit higher oxygen affinities than *Nitrosomonas europaea*, suggesting an advantage under oxygen-limited conditions (Ren *et al.*, 2020). Likewise, AOA generally have higher oxygen affinities than AOB, enabling them to outcompete AOB in low-DO niches (Ren *et al.*, 2020). Although the oxygen affinities of comammox remain unmeasured, genomic and theoretical evidence suggests their potential adaptation to low DO (Lawson and Lücker, 2018). In addition to oxygen, temperature has a significant impact on microbial community structures and activities (Zhou *et al.*, 2018). Previous studies revealed an increased prevalence/occurrence of AOA in tropical WWTPs (>25°C) (Limpiyakorn *et al.*, 2011; Dueholm *et al.*, 2022). Additionally, earlier surveys on microbial communities in tropical municipal WWTPs did not detect comammox *Nitrospira* (Song *et al.*, 2021; Gu *et al.*, 2022). WWTPs in tropical regions, such as Malaysia, may be a novel ecological niche and are expected to harbor distinct nitrifying communities from those found in temperate regions. However, previous studies mostly focused on WWTPs in temperate regions (Saunders *et al.*, 2016), and limited information is currently available on the microbial community structure of nitrifiers and their dynamics in tropical regions. Therefore, comprehensive insights into the composition and dynamics of nitrifying communities in WWTPs in tropical regions are vital.

Based on these findings, the present study investigated the abundance and composition of nitrifiers in two tropical municipal WWTPs in Malaysia through a seven-month sampling campaign. Samples were collected from two full-scale municipal WWTPs in Malaysia operated under high-DO (2.0–5.5‍ ‍mg O_2_ L^–1^) or low-DO (0.2–0.7‍ ‍mg O_2_ L^–1^) conditions at 30°C. Comparisons of nitrifying communities between the high-DO and low-DO processes will provide important information on the nitrifying communities of tropical WWTPs. Microbial communities in these WWTPs were investigated using 16S rRNA gene amplicon sequencing, and nitrifying communities were further exami­ned using a combination of 16S rRNA-targeted fluorescence *in situ* hybridization (FISH) and quantitative PCR (qPCR) ana­lyses targeting AOB, AOA, and comammox *amoA* genes encoding ammonia monooxygenase subunit A. The present results will advance our understanding of the roles these nitrifiers play in biological nutrient removal (BNR) processes in tropical municipal wastewater treatment systems.

## Materials and Methods

### Collection of activated sludge and wastewater samples

Between October 2023 and April 2024, activated sludge samples were collected biweekly from two WWTPs located in Selangor, Malaysia (denoted as WWTP-A and WWTP-B). WWTPs were operated as sequencing batch reactors (SBRs) with a 4-h operational cycle comprising 2‍ ‍h of filling and aeration, 1‍ ‍h of settling, and 1‍ ‍h of decanting. In WWTP-A, the SBR was operated under low-DO conditions, maintaining DO concentrations between 0.2 and 0.7‍ ‍mg O_2_ L^–1^ during the aeration phase (referred to as WWTP-A_lowDO_). In WWTP-B, two SBRs were operated in parallel: one under low-DO conditions (0.2–0.7‍ ‍mg O_2_ L^–1^) and the other under high-DO conditions (2.0–5.5‍ ‍mg O_2_ L^–1^), hereinafter referred to as WWTP-B_lowDO_ and WWTP-B_highDO_, respectively. Water temperatures in sewage were 30±0.6 and 30±0.5°C in WWTP-A and WWTP-B, respectively, and remained stable throughout the sampling period. Detailed operating conditions are shown in Table 1. Activated sludge samples were collected during the aeration phase and transported to the laboratory at 4°C within 2 h.

### Chemical ana­lysis

Mixed liquor samples were subjected to total suspended solids (TSS) and volatile suspended solids (VSS) ana­lyses according to standard methods (APHA, 1998). The biomass concentration (mg‍ ‍dry‍ ‍L^–1^) was measured as the VSS concentration in mixed liquor (*i.e.*, MLVSS). After filtration through a 0.2-μm regenerated cellulose membrane (AF0-2203-52; Phenomenex), nitrite (NO_2_^–^), nitrate (NO_3_^–^), and ammonium (NH_4_^+^) concentrations were assessed by an 861 Advanced Compact IC system (Metrohm). Regarding total nitrogen (TN), samples were filtered through a 0.45-μm cellulose acetate membrane (AF0-8104-52; Minisart^®^, Sartorius) and analyzed using a TOC-V CSN analyzer (Shimadzu) with a TNM-1 module. Chemical oxygen demand (COD_Cr_) was measured with a Hach test kit and DRB 200 digester.

### DNA extraction and 16S rRNA gene amplicon sequencing

Genomic DNA was extracted using a NucleoSpin Soil DNA Extraction Kit (Macherey-Nagel) following the manufacturer’s instructions. The V4 region of the prokaryotic 16S rRNA gene was‍ ‍amplified by a polymerase chain reaction (PCR) using the oligonucleotide primers 515F_ext (5′-GTGYCAGMMGBNKCGGTVA-3′) and 806R-CPR-HD (5′-RGACTAMNVRGGTHTCTAAT-3′), which were modified from Hu *et al.* (2024) to increase the coverage of archaea. The PCR amplicons were sequenced on an Illumina MiSeq (v3 chemistry, 2×300 bp; Illumina) and processed by the Joint Microbiome Facility of the Medical University of Vienna and the University of Vienna (project ID JMF-2404-17) as previously described (Pjevac *et al.*, 2021). Briefly, the DADA2 R package v.1.20.00 (R 4.1.1) was used to infer amplicon sequence variants (ASVs), and the phylogenetic affiliations of the detected ASVs were exami­ned using the SILVA database taxonomy (v.138.1). The sequences of the ASVs affiliated with putative AOB, AOA, NOB, and comammox bacteria were aligned with reference sequences retrieved from the Genbank database to construct phylogenetic trees based on the Maximum Likelihood method and Tamura-Nei mode using MEGA v.11 (Tamura *et al.*, 2021).

### qPCR

qPCR assays for the detection of archaeal, betaproteobacterial, and comammox *amoA* genes were conducted using the oligo­nucleotide primers Arch-amoA-104F/616R (Tourna *et al.*, 2011), amoA-1F/2R (Rotthauwe *et al.*, 1997), and ComA-244F/659R (Pjevac *et al.*, 2017), respectively (Table S3). A CFX96 Real-Time PCR Detection System (Bio-Rad) was used for qPCR assays. The reaction mixture (20‍ ‍μL per tube) contained 1×iQ^TM^ SYBR^®^ Green SuperMix (Bio-Rad), 1–20‍ ‍ng of genomic DNA, and 1.0, 0.4, or 0.4‍ ‍μM of the archaeal, bacterial, or comammox *amoA*-targeted primers, respectively. Assays were performed in duplicate. The specific amplification of the target genes from genomic DNA samples was confirmed through a melting curve ana­lysis and/or agarose gel electrophoresis. Triplicate standard series were generated by ten-fold serial dilutions (10^1^–10^8^ gene copies μL^–1^). Dilution series of plasmid DNA containing betaproteobacterial or comammox *amoA* genes were used for standard curve generation, while the archaeal *amoA* gene used for standard curve generation was obtained from genomic DNA extracted from a pure culture of *Nitrososphaera gargensis*. The correlation coefficient for each of the external standard curves was ≥0.97. The amplification efficiencies of betaproteobacterial, comammox, and archaeal *amoA* genes were 87.1, 80.7, and 111.4%, respectively.

### FISH ana­lysis

Sludge samples were collected from WWTP-A on March 26, 2024 and from WWTP-B on March 21, 2024. Samples were fixed in a 3% (v/v) paraformaldehyde solution at 4°C for 3 h. The hybridization of oligonucleotide probes was performed based on the protocol described by Nielsen and Daims (2009). The following probes were used in the present study: Thaum726 (5′-GCT TTC ATC CCT CAC CGT C-3′) mixed with unlabeled competitor probes for the detection of *Thaumarchaeota* (with 25% formamide [FA] in the hybridization buffer) (Beam, 2015); Arch915 (5′-GTG CTC CCC CGC CAA TTC CT-3′) for most archaea (25% FA) (Stahl and Amann, 1991); Nso1225 (5′-CGC CAT TGT ATT ACG TGT GA-3′), NEU (5′-CCC CTC TGC TGC ACT CTA-3′) mixed with unlabeled competitor probes, and 6a192 (5′-CTT TCG ATC CCC TAC TTT CC-3′) mixed with unlabeled competitor probes for AOB (35% FA) (Mobarry *et al.*, 1996; Adamczyk *et al.*, 2003); Ntspa662 (5′-GGA ATT CCG CGC TCC TCT-3′) and Ntspa712 (5′-CGC CTT CGC CAC CGG CCT TCC-3′) mixed with unlabeled competitor probes for *Nitrospira* spp. (35% FA) (Daims *et al.*, 2001a), and EUB338I-III for most bacteria (Amann *et al.*, 1990; Daims *et al.*, 1999). The above oligonucleotide probes were 5' labeled with the Fluo-3, Cy3, or Cy5 dye. Briefly, for bacterial nitrifiers, probes targeting AOB were labeled with Cy3 (orange fluorescence), those targeting *Nitrospira* were labeled with Fluo-3 (green fluorescence), and EUB338I-III were labeled with Cy5 (red fluorescence). Regarding archaeal nitrifiers, probes targeting archaea were labeled with Cy3, those targeting AOA with Fluo-3, and EUB338I-III were labeled with‍ ‍Cy5. Slides were mounted using the antifading reagent, Vectashield (Vectashield Laboratories) before the examination using an AxioImager 2 Epifluorescence Microscope (Zeiss).

### Data availability

16S rRNA gene amplicon sequencing data have been deposited at the Sequence Read Archive under the BioProject accession PRJNA1223683.

## Results

### Process performance of two full-scale municipal WWTPs

The performance of COD_Cr_, NH_4_^+^-N, and TN removal was exami­ned in WWTP-A and WWTP-B in Malaysia. The chemical composition (*e.g.*, NH_4_^+^-N and TN concentrations) of sewage was similar between WWTP-A and WWTP-B, except for COD concentrations (Table 2, S1, and S2). COD_Cr_, NH_4_^+^-N, and TN removal efficiencies during the sampling campaign were similar among the three reactors (Table 2, Fig. S1, S2, and S3). NH_4_^+^ concentrations in the effluents ranged between 1.0 and 2.9‍ ‍mg NH_4_^+^-N L^–1^ (Table 2), which complied with Malaysia’s discharge standard (A) in the environmental quality regulations 2009 (*i.e.*, 5‍ ‍mg NH_4_^+^-N L^–1^ for sewage) (Department of Environment Malaysia, 2009).

### Community structure of nitrifying bacteria and archaea

The microbial community structure was exami­ned using a 16S rRNA gene amplicon sequence ana­lysis. Dominant ASVs were affiliated with the bacterial phyla *Patescibacteria*, *Bacteroidota*, and *Proteobacteria* (Fig. S4). The 20 most abundant microbial lineages are shown in Fig. S5. *Candidatus* Roizmanbacteria was the dominant bacterial lineage (up to 30.9% relative abundance).

**Fig. 1.**
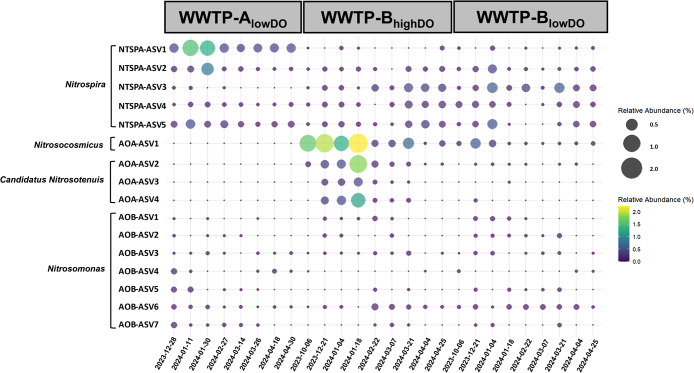
Aerobic ammonia-oxidizing bacteria (AOB), ammonia-oxidizing archaea (AOA), nitrite-oxidizing bacteria (NOB), and putative complete ammonia-oxidizing (comammox, CMX) bacteria detectable by a 16S rRNA gene sequencing ana­lysis in two tropical full-scale WWTPs. The color scale and bubble size denote the relative abundance of each taxon in %.

Seven *Nitrosomonas* ASVs (in the bacterial phylum *Proteobacteria*) represented known AOB in the two WWTPs (Fig. 1), with a *Nitrosomonas oligotropha*-related ASV (AOB-ASV6) (Fig. S6A) being widely distributed across the exami­ned samples. AOB other than *Nitrosomonas* (*e.g.*, *Nitrosospira* and *Nitrosococcus*) were not detected. As‍ ‍for AOA, *Nitrosocosmicus*-related (AOA-ASV1) and *Candidatus* Nitrosotenuis-related ASVs (AOA-ASV2, AOA-ASV3, and AOA-ASV4) (in the archaeal phylum *Nitrososphaerota*) were detected (Fig. S6B). AOA ASVs were more abundant in WWTP-B operated under high-DO conditions (*e.g.*, AOA-ASV1 up to 2.2%) (Fig. 1 and S6D).

The bacterial phylum *Nitrospirota* includes both NOB and comammox bacteria, and five *Nitrospira* ASVs were detected (Fig. S6C). Canonical NOB other than *Nitrospira*, such as *Nitrobacter* and *Nitrotoga* (Daims *et al.*, 2016), were not detected in the present study. *Nitrospira* NTSPA-ASV2 was commonly detected in WWTP-A and WWTP-B, whereas *Nitrospira* NTSPA-ASV1 and NTSPA-ASV3 showed site-specific distributions in WWTP-A and WWTP-B, respectively (Fig. 1).

### Abundance of AOB, AOA, and comammox

**Fig. 2.**
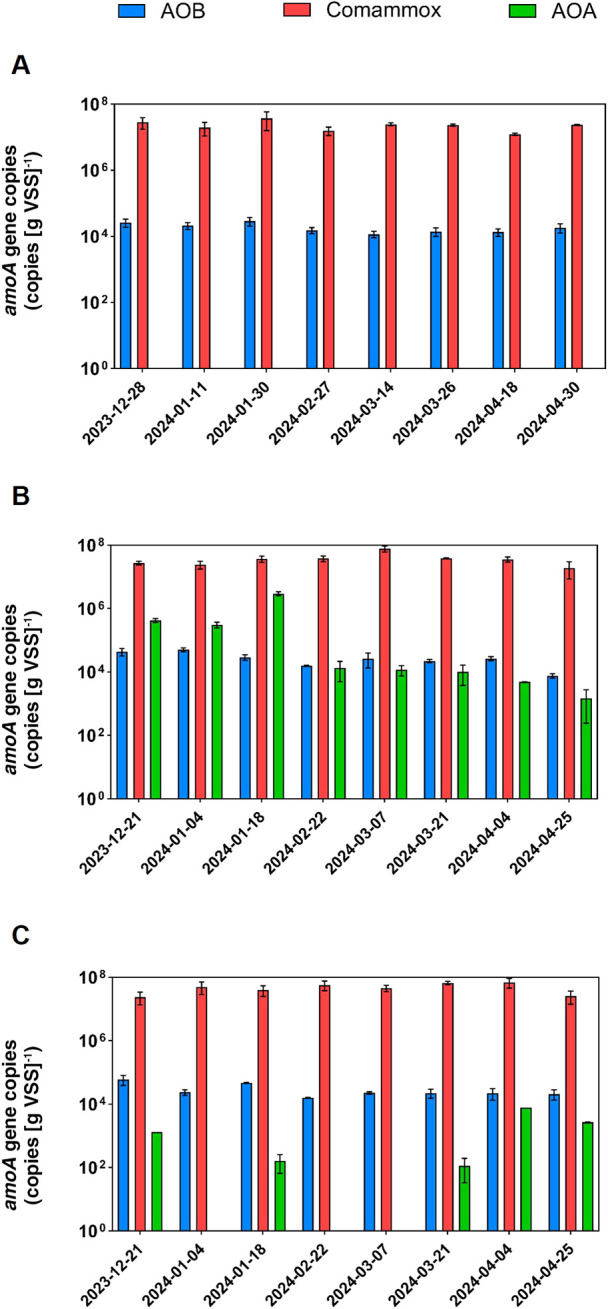
qPCR results showing the *amoA* gene abundance of AOB, comammox *Nitrospira*, and AOA in **(A)** WWTP-A_lowDO_, **(B)** WWTP-B_highDO_, and **(C)** WWTP-B_lowDO_.

qPCR assays targeting the *amoA* genes of AOB, AOA, and comammox were performed to quantify the abundance of these nitrifying bacteria and archaea (Fig. 2). AOB and comammox *amoA* genes were both detected by qPCR in WWTP-A and WWTP-B, with the copy numbers of comammox *amoA* genes (1.22 to 7.77×10^7^ copies [g VSS]^–1^) being three to four orders of magnitude higher than those of AOB *amoA* genes (7.61×10^3^ to 5.95×10^4^ copies [g VSS]^–1^). No *amoA* genes of AOA were detected in WWTP-A, whereas they were present in some samples from WWTP-B_lowDO_ at a lower abundance than AOB and comammox *amoA* genes (up to 2.68×10^3^ copies [g‍ ‍VSS]^–1^; Fig. 2B). AOA were a stable component of the nitrifier community in the reactor WWTP-B_highDO_, where their abundance was similar to or greater than that of AOB (up to 2.97×10^6^ copies [g‍ ‍VSS]^–1^; Fig. 2C).

### Spatial distribution of nitrifying microorganisms in activated sludge

**Fig. 3.**
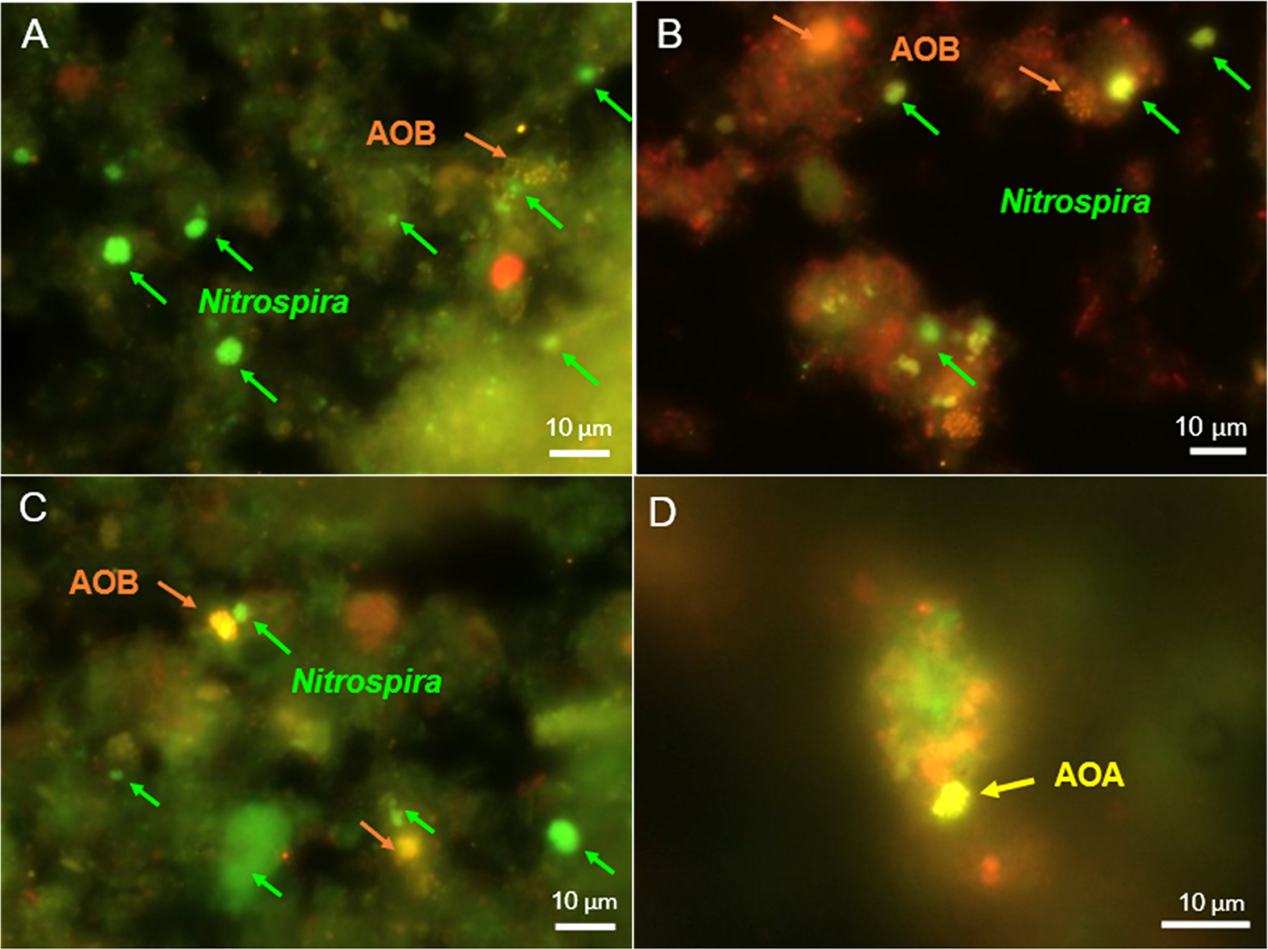
FISH images of activated sludge from **(A)** WWTP-A_lowDO_, **(B)** WWTP-B_lowDO_, and **(C, D)** WWTP-B_highDO_. Sludge samples were collected from WWTP-A on March 26, 2024 and from WWTP-B on March 21, 2024. Bacteria hybridized with the EUB338I-III probe (red) represent the total bacterial population. **(A, B, and C)** Cell aggregates showing orange and green fluorescence represent AOB (probes NSO1225, NEU, and 6a192) and *Nitrospira* (NOB and comammox), respectively (probes Ntspa662 and Ntspa712, covering both canonical and comammox *Nitrospira*). **(D)** WWTP-B_highDO_ hybridized to the probes Thaum726 and Arch915 for the detection of AOA (yellow fluorescence).

A FISH ana­lysis was conducted to further examine the presence of AOA, AOB, and *Nitrospira* bacteria in activated sludge. In all activated sludge samples analyzed, the cell aggregates formed by betaproteobacterial AOB and (comammox or NOB) *Nitrospira* bacteria frequently co-localized in activated sludge flocs (Fig. 3A, B, and C). Additionally, AOA cell clusters were detected in WWTP-B_highDO_ samples (Fig. 3D), consistent with the detection of AOA by 16S rRNA gene amplicon sequencing (Fig. 1) and *amoA*-targeted qPCR (Fig. 2).

## Discussion

### Nitrifying microorganisms in tropical full-scale WWTPs

The present study investigated the community structure and abundance of AOB, AOA, NOB, and comammox bacteria in two WWTPs in Malaysia. The community structure and/or abundance of nitrifying microorganisms in tropical WWTPs have already been exami­ned (Table 3); however, previous studies primarily employed qPCR or a 16S rRNA gene amplicon sequencing ana­lysis, and a comprehensive ana­lysis combining these methods with a FISH ana­lysis has yet to be conducted.

The combination of qPCR, amplicon sequencing, and FISH ana­lyses in the present study allowed us to obtain a more comprehensive understanding of the community structure and abundance of AOB, AOA, NOB, and comammox bacteria. For example, the abundance of comammox *amoA* genes identified by qPCR was three orders of magnitude higher than that of AOB *amoA* genes (Fig. 2). This result was supported by amplicon sequencing data, showing that the relative abundance of lineage II *Nitrospira* (*e.g.*, NTSPA-ASV1, -ASV4, and -ASV5) consistently exceeded that of AOB across all three reactors. However, the magnitude of this difference was less pronounced in amplicon sequencing data than in qPCR, which may be due to PCR biases (Pan *et al.*, 2018).

In contrast, FISH informs on the spatial localization of targeted organisms. Canonical nitrifiers typically co-localize in activated sludge and biofilms, reflecting their mutualistic symbiosis (Daims *et al.*, 2016). Co-localization was observed by FISH for AOB, AOA, and some *Nitrospira* cell clusters (putative NOB), supporting their functional roles as nitrifiers in WWTPs (Fig. 3). *Nitrospira* cell clusters not closely located to AOB or AOA may be comammox organisms, which are independent of other nitrifiers (Fig. 3), although rRNA-based techniques alone (including FISH) do not reliably distinguish comammox from canonical NOB *Nitrospira* (Daims *et al.*, 2015). Furthermore, FISH confirmed the *in situ* presence of AOA, AOB, and *Nitrospira* populations in activated sludge during the sampling period, suggesting that PCR- and sequencing-based methods did not merely detect allochthonous cells (*e.g.*, from the sewage influent) or naked DNA. The applied combination of different mole­cular approaches revealed a complex nitrifying community structure composed of AOA, AOB, and comammox or NOB *Nitrospira* (Fig. 1, 2, and 3). This high nitrifier diversity may reflect the characteristics of sewage, which consists of a complex matrix of organic and inorganic substances, as well as the nature of the bioreactors (*i.e.*, the WWTPs exami­ned were operated as SBRs). In an SBR, COD_Cr_ and NH_4_^+^ concentrations decrease over time, and heterogeneous environments support the growth of physiologically diverse nitrifiers (Siripong and Rittmann, 2007).

### AOB and AOA

*Nitrosomonas* are well-known AOB and are frequently detected as the dominant canonical ammonia oxidizers in WWTPs in temperate and tropical climates (Daims *et al.*, 2001b; Limpiyakorn *et al.*, 2011; Gu *et al.*, 2022). In the present study, phylogenetically diverse *Nitrosomonas* ASVs related to *N. oligotropha*, *N. ureae*, and *N. nitrosa/N. communis* were found in the tropical municipal WWTPs analyzed. Among them, the *N. oligotropha*-related ASV (AOB-ASV6) was the most consistently detected across different reactors and time points (Fig. S6). The coexistence of these phylogenetically distinct *Nitrosomonas* suggests functional diversity and potential niche differentiation among AOB populations. For example, *N. ureae* is considered to be oligotrophic, thriving in environments with low NH_4_^+^ levels (1–5‍ ‍mM), whereas *N. communis* is typically eutrophic, preferring higher NH_4_^+^ concentrations (10–50‍ ‍mM) and elevated oxygen levels (Prosser *et al.*, 2014; Zheng *et al.*, 2022). *N. oligotropha* was the most dominant in our tropical systems. However, this may not be directly attributable to temperature because this lineage has also been frequently reported in temperate WWTPs (Daims *et al.*, 2001b). The characterized *N. oligotropha* strains showed a high substrate affinity for ammonia (*K_s_* for NH_3_; 1.9–4.2‍ ‍μM) (Koops and Pommerening-Röser, 2001) and, thus, are generally present in systems with low concentrations of ammonia. This physiological trait may provide a competitive advantage under low-NH_4_^+^ conditions, which may be common in the studied systems.

In contrast to AOB, AOA do not generally appear to be‍ ‍the key nitrifiers in WWTPs in temperate regions. Conversely, a global study detected AOA in tropical regions, such as Malaysia and the Philippines (Dueholm *et al.*, 2022), and they have also been reported in Thailand and Singapore (Limpiyakorn *et al.*, 2011; Zhang *et al.*, 2011). One potential factor shaping the ecological niche of AOA may be temperature. Previous studies demonstrated that AOA abundance correlated with increased temperatures (22–30°C) (Roy *et al.*, 2017). The temperature effect was more pronounced for AOA than for AOB. This is consistent with the findings of Sauder *et al.* (2012) showing seasonal variations in AOA *amoA* gene abundance in Canada, with the highest levels in September (warmer season) and the lowest levels in February (colder season). Furthermore, Zeng *et al.* (2014) exami­ned the effects of temperature on AOA at 15, 25, and 35°C in China, and revealed that higher temperatures significantly increased archaeal *amoA* gene abundance. Similarly, Wu *et al.* (2013) observed the significant autotrophic growth of AOA in freshwater sediments at 37°C. Therefore, a positive temperature effect may support the growth of AOA in tropical WWTPs. Previous studies highlighted the predominance of AOA over AOB in some municipal systems; *e.g.*, the abundance of AOA *amoA* genes was one to two orders of magnitude greater than that of AOB *amoA* genes in two of the four municipal WWTPs in Thailand (Limpiyakorn *et al.*, 2011). The present study also detected AOA in WWTP-B. 16S rRNA gene sequencing results revealed the presence of several AOA-related ASVs, including one affiliated with *Nitrosocosmicus* (AOA-ASV1) and three affiliated with *Candidatus* Nitrosotenuis (AOA-ASV2, AOA-ASV3, and AOA-ASV4). *Nitrosocosmicus* and *Candidatus* Nitrosotenuis-related AOA generally thrive in warm environments (Sauder *et al.*, 2017; Sauder *et al.*, 2018), suggesting that they adapt well to tropical wastewater conditions. Among them, the abundance of *Nitrosocosmicus*-related AOA-ASV1 increased by up to 2.2% in the WWTP-B_highDO_ reactor (Fig. 1 and S6D). However, the temporal variation in AOA abundance may have been the result of multiple factors that are difficult to generalize, such as sewage characteristics and the spatial structure of sludge flocs or biofilms. A key feature of AOA is their use of a modified hydroxypropionate/hydroxybutyrate (HP/HB) cycle for carbon fixation, which allows them to thrive under extremely low concentrations of electron donors and acceptors. The dominance of AOA over AOB was only observed in WWTP-B_highDO_. This may be due to reduced DO levels in deeper regions or to AOA typically forming compact microcolonies, which may generate oxygen-limited niches within aggregates. Furthermore, the site-specific distribution of AOA in WWTPs has also been reported by Limpiyakorn *et al.* (2011), indicating that AOA have a narrow ecological niche in WWTPs. One potential factor shaping the ecological niche of AOA is the COD_Cr_ concentration in sewage. The COD_Cr_ concentration in sewage was markedly lower in‍ ‍WWTP-B than in WWTP-A (176.6±75.8 and 291.7±78.7‍ ‍mg COD_Cr_ L^–1^, respectively) (Table 2). Organic compounds with a high metal complexation potential have been shown to reduce the bioavailability of copper and, thus, inhibit AOA (Gwak *et al.*, 2020), the electron transport system of which heavily depends on copper (Walker *et al.*, 2010). The lower COD_Cr_ in WWTP-B may be beneficial for the AOA populations detected in this plant. However, the operational conditions in WWTP-B_lowDO_ (>30°C, 177‍ ‍mg COD L^–1^, and 0.2–0.7‍ ‍mg O_2_ L^–1^) may support the activity of sulfate reducers causing the formation of hydrogen sulfide, which easily reacts with soluble copper and decreases copper bioavailability (Shafiee *et al.*, 2021). This may explain why the AOA populations were less stable in WWTP-B_lowDO_ than in WWTP-B_highDO_ (Fig. 2).

Notably, *Nitrosomonas*-related AOB ASVs exhibited very low relative abundance (<0.1 to 0.2%) in samples from WWTP-A_lowDO_ and AOA-related ASVs were not detected in this reactor (Fig. 1), although WWTP-A_lowDO_ showed NH_4_^+^ removal efficiencies of 85±18% during the sampling campaign (Table 2). The population size of AOB/AOA in activated sludge may be roughly estimated using the nitrification model developed by Rittmann *et al.* (1999), which relates the NH_4_^+^ consumption rate to the biomass of nitrifiers. Based on this model, the estimated AOB cell number was approximately 10^8^ cells [g VSS]^–1^, which was five orders of magnitude lower than the number quantified by qPCR (See Supplementary Information [SI] text for details of the calculation). This difference suggests the involvement of other NH_4_^+^-oxidizing microbes (*e.g.*, comammox bacteria) that may be key players in WWTP-A_lowDO_.

### Comammox and NOB Nitrospira

It is important to note that previous surveys on tropical municipal WWTPs (Table 3) did not detect comammox *Nitrospira*. They were either conducted prior to the discovery of comammox (in 2015) or only analyzed the 16S rRNA genes of nitrifiers. All currently known comammox *Nitrospira* belong to *Nitrospira* lineage II, which also contains canonical nitrite-oxidizing *Nitrospira* (NOB). Since comammox and NOB *Nitrospira* mingle in the 16S rRNA gene-based phylogenies of lineage II, they cannot reliably be distinguished by analyzing their 16S rRNA gene sequences (Daims *et al.*, 2015; Van Kessel *et al.*, 2015). Nevertheless, based on current knowledge of the comammox phylogeny, the presence of any 16S rRNA gene ASVs from *Nitrospira* lineage II in our dataset (Fig. S6C) was considered a prerequisite for the potential presence of comammox *Nitrospira* in WWTPs.

Our 16S rRNA gene amplicon sequencing ana­lysis showed a wide distribution of the 2 putative comammox *Nitrospira* ASVs (NTSPA-ASV4 and -ASV5) in plants. The occurrence of comammox *Nitrospira* in the reactors was confirmed by qPCR assays targeting comammox *amoA* genes as a specific functional marker for comammox (Daims *et al.*, 2015; Van Kessel *et al.*, 2015; Pjevac *et al.*, 2017). This approach detected comammox *Nitrospira* under both low-DO and high-DO conditions (Fig. 2). Although *Nitrospira* are aerobic autotrophs, they utilize the oxygen-sensitive reductive tricarboxylic acid cycle for CO_2_ fixation (Lücker *et al.*, 2010), and many NOB and comammox *Nitrospira* lack canonical defense mechanisms against reactive oxygen species (Lücker *et al.*, 2010; Kits *et al.*, 2017). Therefore, *Nitrospira* likely prefer microoxic niches (Lücker *et al.*, 2010), and are often detected in WWTPs operated under low-DO conditions (How *et al.*, 2018; Roots *et al.*, 2019; Yang *et al.*, 2020). It remains unclear whether *Nitrospira* populations thriving in fully aerated systems (Zhao *et al.*, 2022), as observed in this study in WWTP-B_highDO_ (Fig. 2), benefit from reduced DO levels in deeper regions of biofilms or larger flocs (Almstrand *et al.*, 2013).

The qPCR quantification of *amoA* genes suggested that comammox *Nitrospira* were the most abundant ammonia oxidizers in all three reactors (Fig. 2). A potential reason for their predominance may be metabolic versatility because their genomes encode hydrogenases and formate dehydrogenases, enabling growth on hydrogen or formate, in addition to ammonia and/or NO_2_^–^ (Koch *et al.*, 2015; Yang *et al.*, 2020; Leung *et al.*, 2022). Kinetic studies demonstrated that comammox *Nitrospira* exhibited an extremely high substrate affinity for ammonia, which exceeded that of AOB and many non-marine AOA (Kits *et al.*, 2017; Sakoula *et al.*, 2021). Despite slower growth, they yield more biomass per mole of ammonia oxidized (Costa *et al.*, 2006; Kits *et al.*, 2017). This aligns with findings from biofilm-rich systems, in which comammox often outnumber other nitrifiers (Zhao *et al.*, 2022). Consistent with a predicted niche in biofilms, comammox *Nitrospira* outnumbered all other nitrifiers in biofilm samples from rotating biological contactors in a municipal WWTP in Canada (Spasov *et al.*, 2020), suggesting that similar niche conditions exist in our SBRs. In addition, comammox have been shown to outcompete AOB under copper-limited conditions (Koike *et al.*, 2022), which may explain their high abundance in our reactors despite non-limiting NH_4_^+^ concentrations. On the other hand, if copper was a limiting factor, the high abundance of AOA in WWTP-B was unexpected (see also the discussion above on AOA abundance). Further investigations, including measurements of copper concentrations, will be required to disentangle the selective factors that facilitate the growth and activity of comammox, AOA, and AOB and also to elucidate how these factors, in combination, shape nitrifier population structures in tropical and temperate WWTPs.

All *Nitrospira* ASVs were affiliated with sublineage I (NTSPA-ASV2 and -ASV3) or sublineage II (NTSPA-ASV1, -ASV4, and -ASV5) (Fig. S6C). Among them, temporal variations in *Nitrospira* abundance were observed despite relatively stable reactor performance (Fig. 1, Table 2). Such variation may be shaped by complex ecological interactions and functional redundancy among coexisting nitrifiers. *Nitrospira* bacteria have distinct ecological niches and those affiliated into sublineages I and II have different genetic potentials (*e.g.*, the presence of urease and group 2a [NiFe] hydrogenase) and physiological traits, including their NO_2_^–^ oxidation capacity and tolerance to free ammonia. In comparisons with lineage I, lineage II retains the core metabolic functions of *Nitrospira*, but shows large genomic differences, including genes associated with adaptation to high oxygen concentrations. Their temporal variation is likely driven by metabolic versatility and reciprocal feeding interactions, particularly their involvement in multiple nitrogen transformation processes. Nevertheless, the physiological characteristics and ecological dynamics of *Nitrospira* in tropical WWTPs remain unclear, and further studies that examine the niche differentiation of these *Nitrospira* bacteria are warranted.

## Conclusion

The present study provides the first insights into the abundance and composition of all currently known groups of nitrifying microorganisms in two tropical municipal WWTPs. The results obtained herein contribute to our understanding of tropical nitrifiers, which may ultimately help optimize sewage treatment processes in tropical climates. For example, the dominance of comammox and AOA over AOB, as observed in WWTP-B_highDO_, may reduce the environmental impact of wastewater treatment. Since both groups exhibit a higher affinity for ammonia than AOB, their activity may lead to lower effluent NH_4_^+^ concentrations. However, a more detailed understanding of the specific ecological niches and relative contributions of AOA, AOB, NOB, and comammox organisms to overall nitrification is needed before targeted measures to control nitrifier populations in tropical WWTPs may be developed.

## Citation

Feng, L., Loi, J. X., Séneca, J., Pjevac, P., Adnan, F. H., Ngoh, G. C., et al. (2025) Nitrifying Communities in Biological Nitrogen Removal Processes at Tropical Municipal Wastewater Treatment Plants. *Microbes Environ ***40**: ME25036.

https://doi.org/10.1264/jsme2.ME25036

## Supplementary Material

Supplementary Material

## Figures and Tables

**Table 1.** Sequencing batch reactors exami­ned in the present study.

	WWTP-A_lowDO_	WWTP-B_lowDO_	WWTP-B_highDO_
DO level (mg DO L^–1^)	0.2–0.7	0.2–0.7	2.0–5.5
Volume (m^3^)	641	452	452
Temperature (°C)	30±0.6	30±0.5	30±0.5
OLR*	480±129	428±184	428±184
ALR*	34.8±8.9	51.2±11.9	51.2±11.9
NLR*	45.9±8.5	60.6±10.9	60.6±10.9
Biomass** (mg dry L^–1^)	2,654±579	1,827±640	2,622±1,258
HRT*	14.6	9.9	9.9
SRT*	15	15	15

* OLR: organic loading rate (kg COD_Cr_ m^–3^ d^–1^); ALR: ammonium loading rate (kg NH_4_^+^ N m^–3^ d^–1^); NLR: total nitrogen loading rate (kg TN m^–3^ d^–1^); HRT: hydraulic retention time (h); SRT: sludge retention time (d). **Biomass: biomass concentration measured as mixed liquor volatile suspended solids (MLVSS).

**Table 2.** Process performance of three full-scale sequencing batch reactors.

		WWTP-A_lowDO_	WWTP-B_lowDO_	WWTP-B_highDO_
NH_4_^+^-N	Influent (mg NH_4_^+^-N L^–1^)	21.2±5.4	21.1±4.9	21.1±4.9
	Effluent (mg NH_4_^+^-N L^–1^)	2.9±2.6	1.5±1.8	1.0±1.7
	Removal efficiency (%)	85±18	93±8	96±6
TN	Influent (mg TN L^–1^)	27.9±5.2	25.0±4.5	25.0±4.5
	Effluent (mg TN L^–1^)	5.8±2.9	6.0±3.3	8.9±4.3
	Removal efficiency (%)	79±11	76±11	64±15
COD_cr_	Influent (mg COD_Cr_ L^–1^)	291.7±78.7	176.6±75.8	176.6±75.8
	Effluent (mg COD_Cr_ L^–1^)	2.6±4.8	6.4±9.3	10.8±13.6
	Removal efficiency (%)	99±2	94±10	92±11

**Table 3.** Nitrifying microbial community in full-scale tropical municipal wastewater treatment plants.

Location	(°C)^a^	Analysis^b^	AOB (*Nitrosomonas*)	AOA	*Nitrospira* NOB/comammox	Reference
Malaysia	30±0.6	qPCR NGS FISH	*N. oligotropha* *N. ureae* *N. nitrosa/communis*	*Nitrosocosmicus*, *Ca.* Nitrosotenuis	*Nitrospira defluvii*, *Nitrospira lenta* *Ca.* Nitrospira nitrificans, *Ca.* Nitrospira nitrosa	This study
	31±1.7	NGS	*Nitrosomonas sp.* (midas_s_139, midas_s_11707, midas_s_11773)	*Nitrososphaeraceae* sp.	*Nitrospira defluvii*, *Nitrospira nitrosa* *Nitrospira* sp. (midas_s_9970, midas_s_11142, midas_s_10386)	(Dueholm *et al.*, 2022)c
Thailand	*n.a.*	qPCR	*N. oligotropha*, *N. europaea*, *Nitrosomonas* sp.	Soil group 1.1b Marine group 1.1a	*n.a.*	(Limpiyakorn *et al.*, 2011)
	>25	NGS	*Nitrosomonas* sp.	*Thaumarchaeota* sp.	*Nitrospira* sp.	(Song *et al.*, 2021)
Singapore	*n.a.*	*qPCR* NGS	*N. ureae* *N. oligotropha*, *Nitrosomonas* sp.	*Thaumarchaeota* sp.	*n.a.*	(Zhang *et al.*, 2011)
	30±0	NGS	*Nitrosomonas sp.* (midas_s_11773)	*n.d.*	*Nitrospira defluvii*, *Nitrospira nitrosa* *Nitrospira* sp. (midas_s_9970)	(Dueholm *et al.*, 2022)c
Southern China	23–32	NGS	*Nitrosomonas* sp.	*n.d.*	*Nitrospira* sp.	(Gu *et al.*, 2022)
Philippines	29±1.8	NGS	*Nitrosomonas sp.* (midas_s_11773)	*Nitrososphaeraceae* sp. *Nitrosopumilaceae* sp.	*Nitrospira defluvii* *Nitrospira nitrosa* *Nitrospira* sp. (midas_s_9970, midas_s_11142, midas_s_10386)	(Dueholm *et al.*, 2022)c

^a^ Temperature of wastewater, ^b^ qPCR assay for the *amoA* gene, NGS; 16S rRNA gene amplicon sequencing ana­lysis, FISH; fluorescence *in situ* hybridization. ^c^ data where the climate zone is “Af” (tropical rainforest) or “Am” (tropical monsoon) and the plant type is “activated sludge” *n.a.*: not available in the manuscript, *n.d.*; not detected
