# Supplementary material for: Nitrifying Communities in Biological Nitrogen Removal Processes at Tropical Municipal Wastewater Treatment Plants
Source: Microbes Environ. 2025 Sep 10;40(3):ME25036. doi: 10.1264/jsme2.ME25036 (PMC12501877; doi:10.1264/jsme2.ME25036)
Supplement: Supplementary file 1 — Supplementary Material [file 40_25036_s1.pdf]

Supplementary Information

Title:

Nitrifying communities in biological nitrogen removal processes at tropical municipal wastewater treatment plants

Authors:

Liang Feng[1,*], Jia Xing Loi[1,*], Joana Séneca[2,3], Petra Pjevac[2,3], Faidzul Hakim Adnan[1], Gek Cheng Ngoh[1], Bee Chin Khor[4], Alijah Mohd Aris[4], Mamoru Oshiki[5], Holger Daims[2,6], Adeline Seak May Chua[1,**]

**These two authors contributed equally to this work.*

Affiliation:

1. Sustainable Process Engineering Center, Department of Chemical Engineering, Faculty of Engineering, Universiti Malaya, 50603 Kuala Lumpur, Malaysia
2. Division of Microbial Ecology, Centre for Microbiology and Environmental Systems Science, University of Vienna, 1030 Vienna, Austria
3. Joint Microbiome Facility of the Medical University of Vienna and the University of Vienna, 1030 Vienna, Austria
4. Indah Water Konsortium Sdn Bhd, No. 1, Jalan Damansara, 60000 Kuala Lumpur, Malaysia
5. Division of Environmental Engineering, Faculty of Engineering, Hokkaido University, 060-8628 Hokkaido, Japan
6. The Comammox Research Platform, University of Vienna, 1030 Vienna, Austria

*Contact Details of Correspondence**:*

Email Address: adeline@um.edu.my (A. S. M. Chua)

SI text

Modeling the Abundance of Ammonia-Oxidizing Microorganisms in WWTP-A_{lowDO}.

The abundance of ammonia oxidizers in the WWTP-A_{lowDO} was estimated from levels of ammonia removal using the model developed by Furumai and Rittmann (1994); Mußmann et al. (2011); Rittmann et al. (1999). Ammonia oxidizer biomass (X_{AO}) was estimated using the following equation:

$$X_{AO} = \frac{\theta x}{\theta}\left[\frac{Y_{AO}}{1+b_{AO}\times\theta x} \times \Delta Ammonia\right] \approx 242.6 \text{ mg VSS/L}$$

Here,

X_{AO}; the biomass of ammonia oxidizers in milligrams per liter

θ_x; the mean cell residence time in days (**Table 1**)

θ; the hydraulic retention time in days (**Table 1**)

Y_{AO}; the growth yield of nitrifiers (1.15 g dw/mol NH_4-N) (Mußmann et al., 2011)

b_{AO}; the endogenous respiration constant of ammonia oxidizers (0.15 d^{-1}) (Mußmann et al., 2011)

ΔAmmonia; the difference in influent and effluent ammonia concentrations in milligrams per liter (**Table 2**).

Assuming an AOB cell volume of 0.75 (Mußmann et al., 2011), and the biomass values obtained from the model were converted to biovolume using a conversion factor of 310 fg·C·μm^3 (Fry, 1990), the cell number of AOB (X'_{AOB}) was calculated as follows:

$$X'_{AOB} = \frac{242.6 \times 10^9 \text{ fg/L}}{0.75 \times 310 \text{ fg}/\mu\text{m}^3} \approx 1.04 \times 10^9 \text{cells/L}$$

Based on the sludge concentration of 2.65 g VSS/L (**Table 1**), the number of cells in AOB ($X''AOB$) was

$$X''_{AOB} = \frac{1.04 \times 10^9 \text{ cells/L}}{2.65 \text{ VSS/L}} \approx 3.94 \times 10^8 \text{cells/L}$$

The abundance of AOB was quantified based on the *amoA* gene copies detected by qPCR, which was around 10^4 *amoA* gene copies per gram of volatile suspended solids. Assuming that each AOB cell contains 1 to 5 *amoA* gene copies (with a median value of 3 copies per cell), the AOB cell number was calculated as follows:

$$\text{AOB cell number} = \frac{10^4 \; amoA \text{ gene copies/g VSS}}{3 \; amoA \text{ gene copies/cell}} \approx 3.33 \times 10^3 \text{ cells/g VSS}$$

The abundance of comammox was quantified based on the *amoA* gene copies detected by qPCR, which was 10^8 gene copies per gram of volatile suspended solids. Assuming

that each comammox cell contains a single *amoA* gene copy, the comammox cell number was calculated as follows:

$$\text{comammox cell number} = \frac{10^7\ amoA\ \text{gene copies/g VSS}}{1\ amoA\ \text{gene copy/cell}} = 10^7\ \text{cells/g VSS}$$

The model-estimated AOB cell number (3.94×10^8 cells/g VSS) was four orders of magnitude higher than the AOB cell number quantified by qPCR (3.33×10^3 cells/g VSS). In contrast, the comammox cell number (10^7 cells/g VSS) was comparable to the model-estimated AOB cell number.

The significant discrepancy between the model-estimated AOB cell number and the qPCR-based quantification suggests that the model may overestimate the abundance of AOB in the system. This overestimation could be attributed to the assumption that AOB are the sole ammonia-oxidizing microorganisms, while comammox, with a cell number of 10^7 cells/g VSS, likely plays a dominant role in ammonia oxidation. The close agreement between the comammox cell number and the model-estimated AOB cell number further supports the hypothesis that comammox is the primary driver of ammonia oxidation in this system.

Supplementary Figures

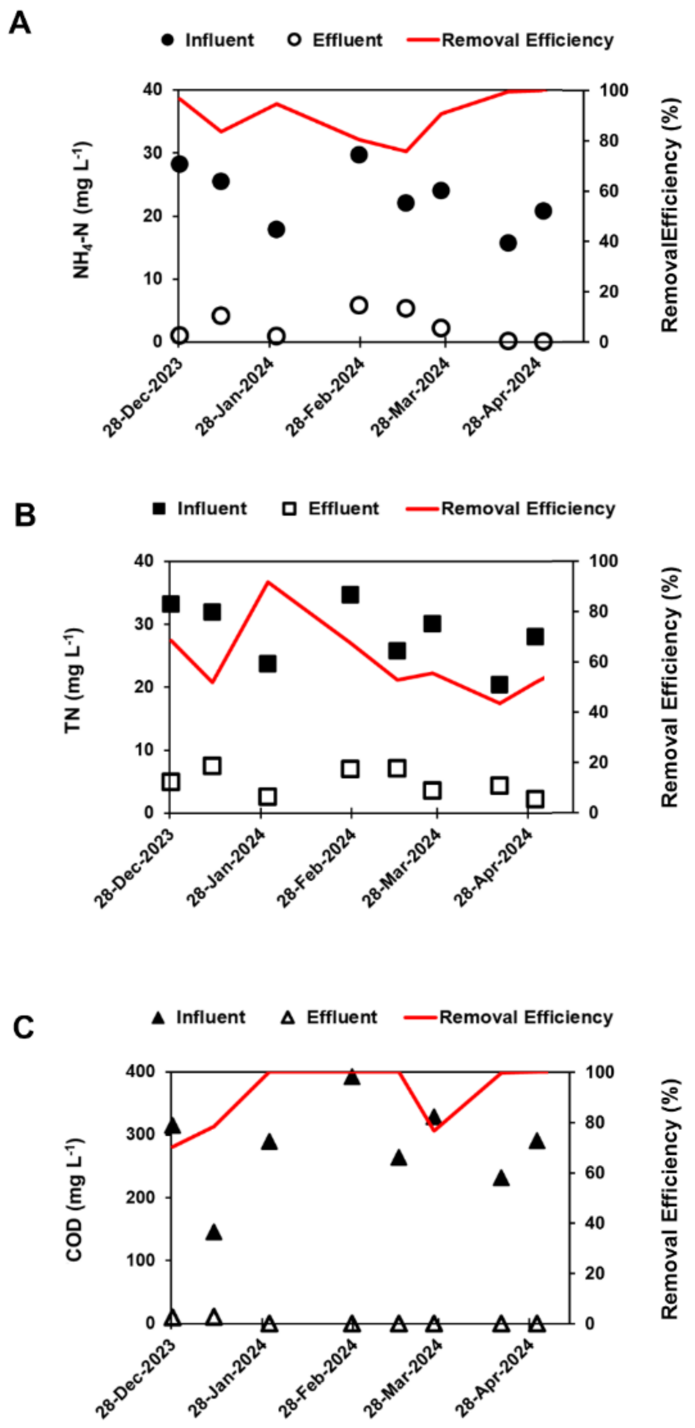

Figure S1. Profiles of (A) NH_4-N, (B) TN and (C) COD_{Cr} in WWTP-A_{lowDO}.

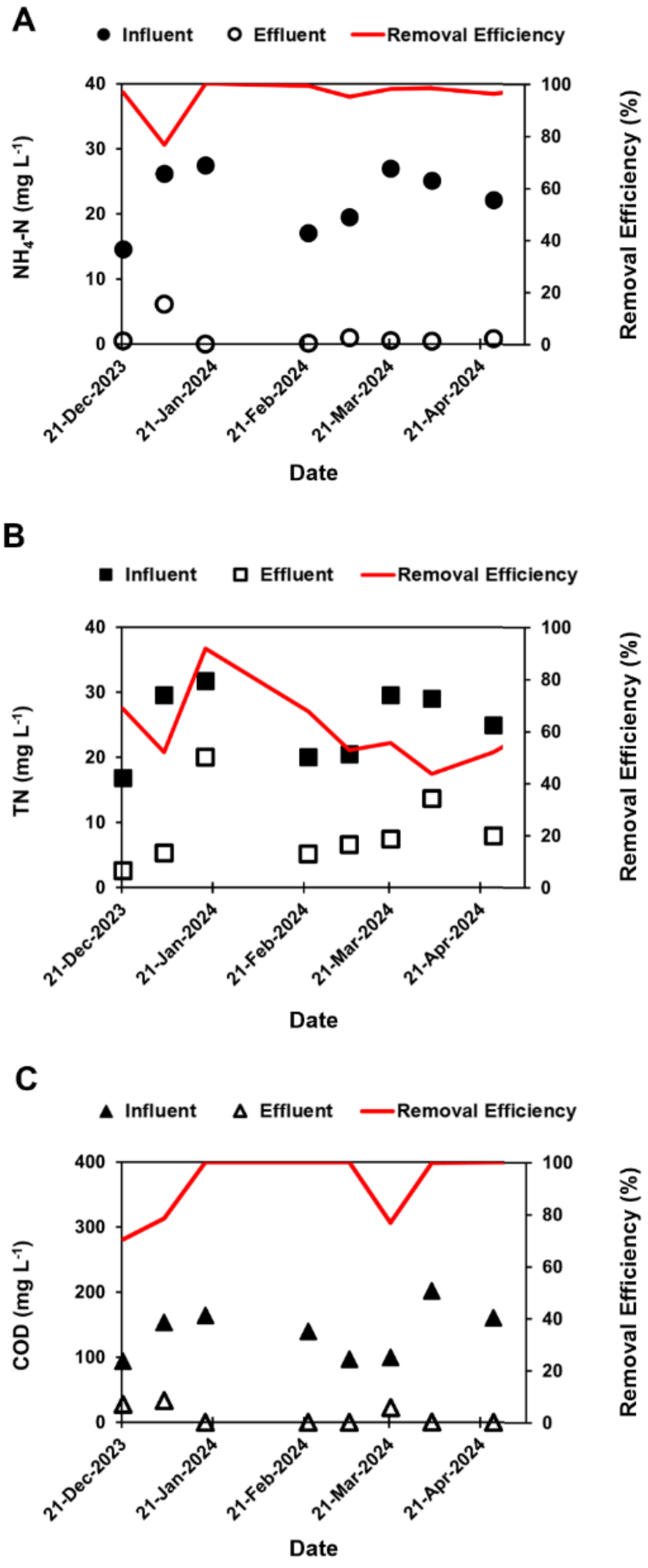

Figure S2. Profiles of (A) NH_4-N, (B) TN and (C) COD_{Cr} in WWTP-B_{highDO}.

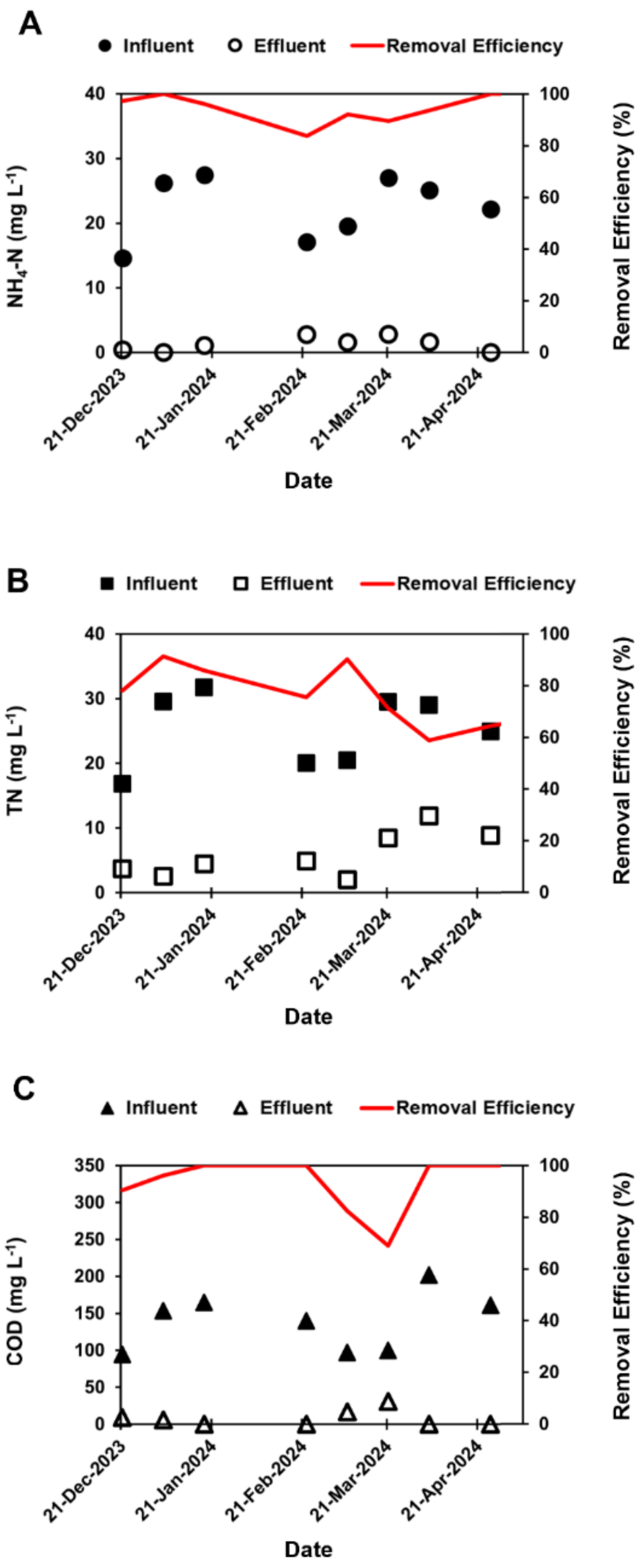

Figure S3. Profiles of (A) NH_4-N, (B) TN and (C) COD_{Cr} in WWTP-B_{lowDO}.

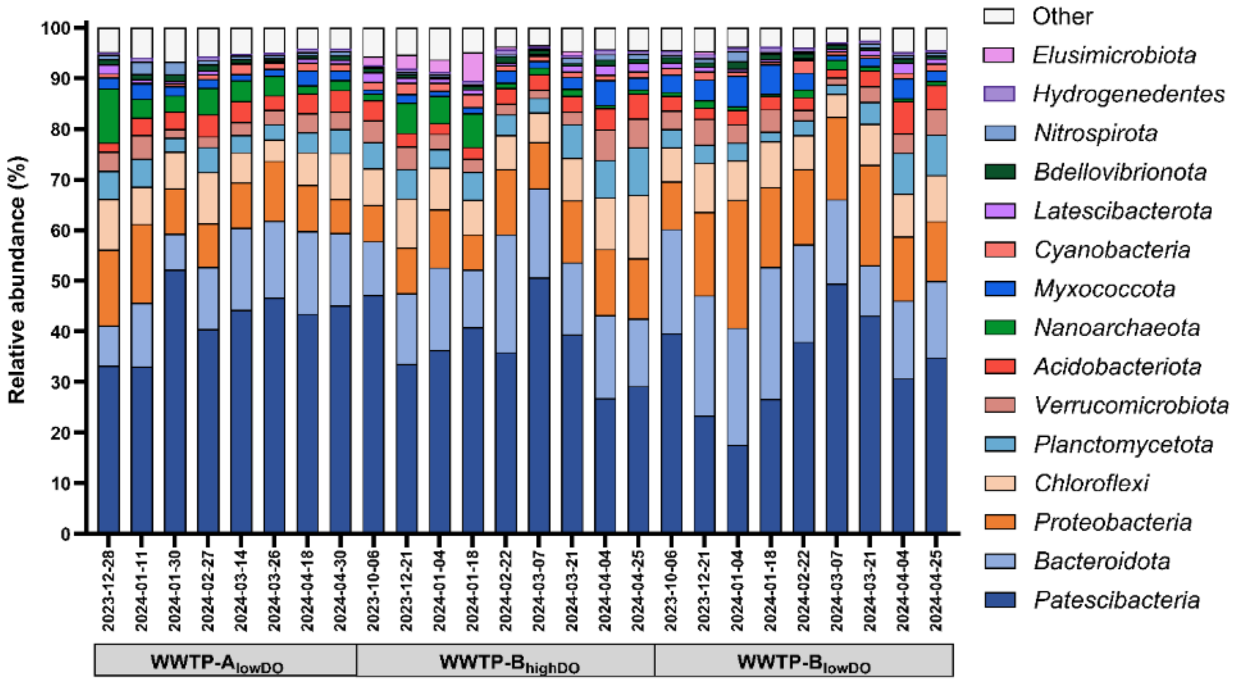

Figure S4. Microbial community structures in the analyzed tropical municipal WWTPs. Bars depict the relative abundances (%) of 16S rRNA gene reads at the phylum level in each activated sludge sample.

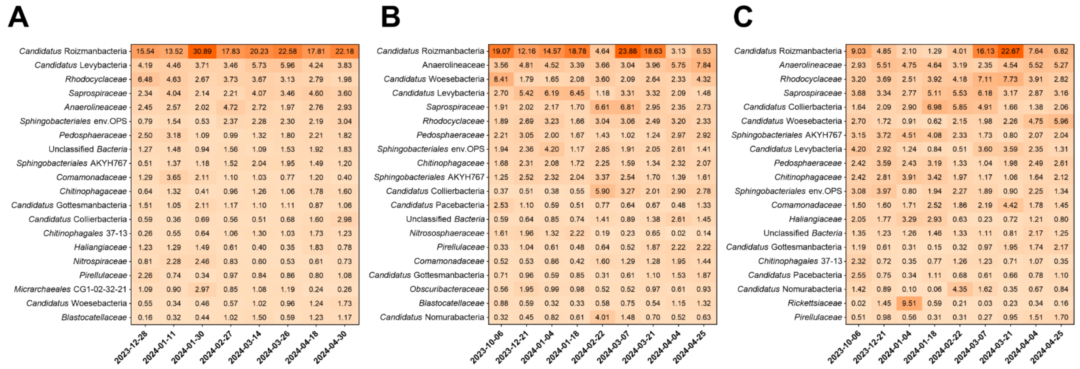

Figure S5. Relative abundances of the 20 most abundant microbial taxa in the WWTP-A and WWTP-B sludge samples. The taxa were identified at the family level. **(A)** WWTP-A_{lowDO}; **(B)** WWTP-B_{highDO}; **(C)** WWTP-B_{lowDO}.

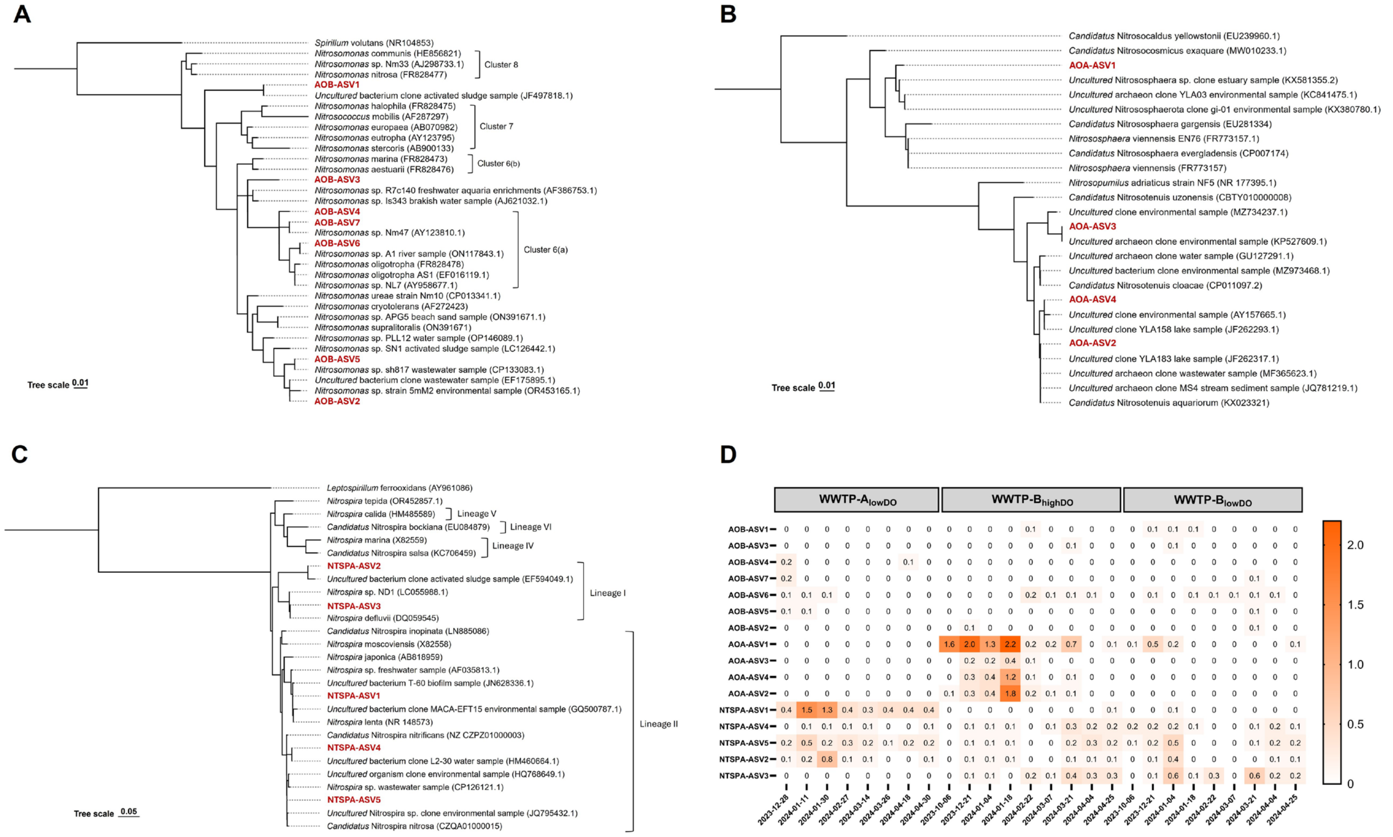

Figure S6. Maximum Likelihood phylogenetic trees based on 16S rRNA gene sequences showing the phylogenetic relationships of the ASVs with reference sequences. All bootstrap values >90%. ASVs obtained in this study are printed in boldface. **(A)** AOB; **(B)** AOA**; (C)** NOB and comammox bacteria. **(D)** Heatmap shows the read abundance (in percent) of each ASV in WWTP-A and WWTP-B.

Supplementary Tables

Table S1. Influent and effluent characteristics of WWTP-A_{lowDO}.

	WWTP-A_{lowDO} Influent (n = 12)				WWTP-A_{lowDO} Effluent (n = 12)			
**Parameter**	**Average**	**Minimum**	**Maximum**	**Standard Deviation**	**Average**	**Minimum**	**Maximum**	**Standard Deviation**
**Temperature (℃)**	30.0	29.1	30.9	0.5	30.3	29.9	30.8	0.4
**pH**	7.1	6.9	7.2	0.1	6.5	6.3	6.7	0.2
**TSS (mg/L)**	83	49	141	24	16	5	31	8
**COD_{cr} (mg/L)**	292	146	417	78	3	0	11	5
**NH_4-N (mg/L)**	21	14	30	5	3	0	9	3
**NO_2-N (mg/L)**	0.02	0.00	0.17	0.05	0.79	0.00	4.46	1.72
**NO_3-N (mg/L)**	0.00	0.00	0.01	0.00	1.81	0.00	11.07	3.25
**TN (mg/L)**	28	20	35	5	6	2	13	3

Table S2. Influent and effluent characteristics of WWTP-B$_{lowDO}$ and WWTP-B$_{highDO}$.

	WWTP-B Influent (n=12)				WWTP-B$_{lowDO}$ Effluent (n = 12)				WWTP-B$_{highDO}$ Effluent (n = 12)			
**Parameter**	**Average**	**Minimum**	**Maximum**	**Standard Deviation**	**Average**	**Minimum**	**Maximum**	**Standard Deviation**	**Average**	**Minimum**	**Maximum**	**Standard Deviation**
**Temperature (°C)**	30.3	29.5	31.2	0.5	30.4	30.1	30.6	0.2	30.3	30.0	30.8	0.3
**pH**	7.0	6.9	7.2	0.1	6.3	5.6	6.8	0.4	6.5	6.1	6.8	0.2
**TSS (mg/L)**	51	16	76	20	14	3	39	11	11	5	26	7
**COD$_{cr}$ (mg/L)**	177	339	94	76	6	0	31	9	11	0	58	14
**NH_4-N (mg/L)**	21	13	30	5	2	0	6	2	1	0	6	2
**NO_2-N (mg/L)**	0.01	0.00	0.12	0.03	0.06	0.00	0.36	0.11	0.30	0.00	3.43	0.99
**NO_3-N (mg/L)**	0.01	0.00	0.03	0.02	2.87	0.04	9.61	3.12	6.02	0.15	12.40	3.63
**TN (mg/L)**	25	17	32	5	6	1	12	3	9	3	16	4

Table S3. qPCR primers used in the present study.

Primer name	Sequence (5′–3′)	Target genes	Reference
Arch-amoA-104F	GCAGGAGACTAYATHTTCTA	AOA *amoA*	(Tourna et al.,
Arch-amoA-616R	GCCATCCATCTRTADGTCCA		2011)
amoA-1F	GGGGTTTCTACTGGTGGT	AOB *amoA*	(Rotthauwe et al.,
amoA-2R	CCCCTCKGSAAAGCCTTCTTC		1997)
ComA-244F	TAYAAYTGGGTSAAYTA	Comammox clade *amoA*	(Pjevac et al.,
ComA-659R	ARATCATSGTGCTRTG		2017)

Reference

Fry, J.C. (1990) *Methods in Microbiology*, pp. 41-85, Elsevier.

Furumai, H. and Rittmann, B.E. 1994. Evaluation of multiple-species biofilm and floc processes using a simplified aggregate model. *Water Science and Technology* 29(10), 439-446

Mußmann, M., Brito, I., Pitcher, A., Sinninghe Damsté, J.S., Hatzenpichler, R., Richter, A., Nielsen, J.L., Nielsen, P.H., Müller, A. and Daims, H. 2011. Thaumarchaeotes abundant in refinery nitrifying sludges express *amoA* but are not obligate autotrophic ammonia oxidizers. *Proceedings of the National Academy of Sciences* 108(40), 16771-16776

Pjevac, P., Schauberger, C., Poghosyan, L., Herbold, C.W., van Kessel, M.A.H.J., Daebeler, A., Steinberger, M., Jetten, M.S.M., Lücker, S., Wagner, M. and Daims, H. 2017. AmoA-Targeted Polymerase Chain Reaction Primers for the Specific Detection and Quantification of Comammox Nitrospira in the Environment. *Frontiers in Microbiology* 8.10.3389/fmicb.2017.01508

Rittmann, B.E., Laspidou, C.S., Flax, J., Stahl, D.A., Urbain, V., Harduin, H., Van Der Waarde, J.J., Geurkink, B., Henssen, M.J. and Brouwer, H. 1999. Molecular and modeling analyses of the structure and function of nitrifying activated sludge. *Water Science and Technology* 39(1), 51-59

Rotthauwe, J.H., Witzel, K.P. and Liesack, W. 1997. The ammonia monooxygenase structural gene amoA as a functional marker: molecular fine-scale analysis of natural ammonia-oxidizing populations. *Applied and Environmental Microbiology* 63(12), 4704-4712.10.1128/aem.63.12.4704-4712.1997

Tourna, M., Stieglmeier, M., Spang, A., Könneke, M., Schintlmeister, A., Urich, T., Engel, M., Schloter, M., Wagner, M., Richter, A. and Schleper, C. 2011. *Nitrososphaera viennensis*, an ammonia oxidizing archaeon from soil. *Proceedings of the National Academy of Sciences* 108(20), 8420-8425.doi:10.1073/pnas.1013488108
